# Database-Guided Discovery of Potent Peptides to Combat HIV-1 or Superbugs

**DOI:** 10.3390/ph6060728

**Published:** 2013-05-27

**Authors:** Guangshun Wang

**Affiliations:** Department of Pathology and Microbiology, University of Nebraska Medical Center, 986495 Nebraska Medical Center, Omaha, NE 68198-6495, USA; E-Mail: gwang@unmc.edu; Tel.: +1-402-559-4176; Fax: +1-402-559-4077

**Keywords:** *ab initio* design, antimicrobial peptides, biofilms, database screening, *de novo* design, HIV-1, improved 2D NMR method, MRSA, superbugs, template-based design

## Abstract

Antimicrobial peptides (AMPs), small host defense proteins, are indispensable for the protection of multicellular organisms such as plants and animals from infection. The number of AMPs discovered per year increased steadily since the 1980s. Over 2,000 natural AMPs from bacteria, protozoa, fungi, plants, and animals have been registered into the antimicrobial peptide database (APD). The majority of these AMPs (>86%) possess 11–50 amino acids with a net charge from 0 to +7 and hydrophobic percentages between 31–70%. This article summarizes peptide discovery on the basis of the APD. The major methods are the linguistic model, database screening, *de novo* design, and template-based design. Using these methods, we identified various potent peptides against human immunodeficiency virus type 1 (HIV-1) or methicillin-resistant *Staphylococcus aureus* (MRSA). While the stepwise designed anti-HIV peptide is disulfide-linked and rich in arginines, the *ab initio* designed anti-MRSA peptide is linear and rich in leucines. Thus, there are different requirements for antiviral and antibacterial peptides, which could kill pathogens via different molecular targets. The biased amino acid composition in the database-designed peptides, or natural peptides such as θ-defensins, requires the use of the improved two-dimensional NMR method for structural determination to avoid the publication of misleading structure and dynamics. In the case of human cathelicidin LL-37, structural determination requires 3D NMR techniques. The high-quality structure of LL-37 provides a solid basis for understanding its interactions with membranes of bacteria and other pathogens. In conclusion, the APD database is a comprehensive platform for storing, classifying, searching, predicting, and designing potent peptides against pathogenic bacteria, viruses, fungi, parasites, and cancer cells.

## 1. Introduction

The identification of antimicrobial peptides (AMPs), small proteins important in host defense, can be traced to the discovery of lysozyme by Alex Fleming in 1921 [1]. Another well-known peptide antibiotics, gramicidin, was also discovered as early as 1939 [2]. Some polypeptides, initially discovered for other purposes or functions, were established as AMPs later on. For example, kalata B1, a typical cyclotide from plants, was identified initially in 1973 to have uterotonic activity [3]. Its antimicrobial activity was not established until 1999 [4]. In the 1980s, Hans Boman identified cecropins in insects [5], Robert Lehrer isolated defensins from humans [6], and Michael Zasloff discovered magainins in frogs [7]. These pioneering discoveries ignited the interest of many other investigators in these host defense peptides, leading to the purification and characterization of hundreds of new AMPs.

The rapid increase in natural AMPs makes it a challenging task to manage such information manually. As a consequence, several databases, including APD [8,9], DAMPD [10], CAMP [11], and YADAMP [12] were constructed to store AMPs from both prokaryotes and eukaryotes. Other databases, including the two earlier databases (AMSDb and peptaibols) [13,14], have a narrower scope. While AMSDb contains only AMPs from eukaryotes [13], others are even more specialized databases for plant AMPs (PhytAMP) [15], shrimp AMPs (PenBase) [16], amphibian peptides (DADP) [17], fungal peptides (peptaibols database) [14], bacteriocins (BACTIBASE and BAGEL) [18,19]. There are also databases for special classes of natural AMPs: bacterial thiopeptides (THIOBASE) [20], circular polypeptides (Cybase) [21], defensins [22], and large lytic proteins (EnzyBase) [23]. In addition, SAPD is an old database with only ~200 synthetic peptides [24] and RAPD is another small database for recombinant AMPs, which may, and may not, correspond to natural sequences [25]. Ideally, newer databases should avoid duplicated efforts and provide complementary features to existing database. For example, CAMP collected 1153 peptides predicted to be AMPs and 1651 peptides from patents [11]. Some information about AMP patents and grants can also be found in Defensins KnowledgeBase [22]. DADP contain sequences for the signal and pro-regions of amphibian peptides, which can have many different functions [17]. Cybase is a collection of all circular proteins (*i.e.*, polypeptides with a peptide bond formation between the N- and C-termini), which can possess various functions such as antimicrobial, insecticidal and anticancer activities [21]. Although not yet demonstrated, YADAMP was created to facilitate QSAR studies of AMPs. These databases are chronically listed in the “links” of the APD and are further described in a recent book chapter [26].

The antimicrobial peptide database (APD) was developed for multiple purposes, including peptide nomenclature, classification, statistics, information search, peptide calculation, prediction and design [8,9,26]. In particular, the pipeline design of the APD enables users to search peptide activity and parameters either individually or in combination. Such a feature facilitates database guided peptide discovery. In the following, we first highlight the selected features of the APD and then describe its applications in peptide identification and design. To our knowledge, the use of other databases [10,11,12,13,14,15,16,17,18,19,20,21,22,23,24,25] in peptide design has not been demonstrated.

## 2. The APD Database Features Useful for Peptide Design

### 2.1. Peptide Sources

**Figure 1:**
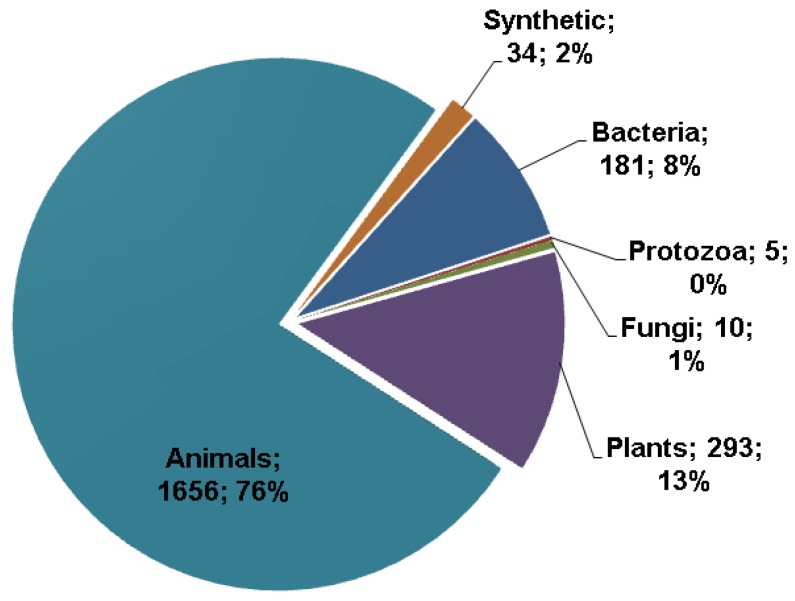
Sources of antimicrobial peptides (in total: 2183) in the antimicrobial peptide database [8,9] in March 2013. The three major sources are animals (76%), plants (13%), and bacteria (8%).

The APD database was initially built in 2003 with 525 entries [8]. These peptide entries were manually collected and registered into the database. Since 2007, this database has been further expanded and regularly updated. The second version of the database reported 1228 AMPs in 2009 [9]. As of March 2013, there were 2183 such peptides. For a natural peptide to be an APD entry, it must have demonstrated antimicrobial activity and known amino acid sequence, at least partially. The sources of the AMPs are annotated, and can be searched, in the name field. In total, there are 1656 AMPs from animals, 293 from plants, 181 from bacteria, five from protozoa, and 10 from fungi (Figure 1). It is clear that the AMPs in the APD originate from a variety of sources and the majority (76%) are identified from animal sources [26], especially amphibians [27,28,29,30,31]. The APD also collected a small number of synthetic peptides (2%). One of the reasons for the small number is that in many cases synthetic peptides are treated as derivatives of natural AMPs and are described under the parent entries. Although this practice reduces the number of AMPs, it does facilitate users to get a more complete view about a particular peptide.

**Figure 2:**
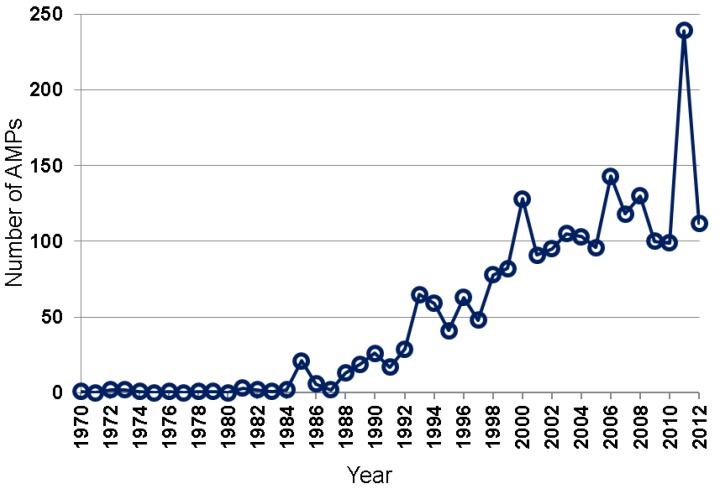
Antimicrobial peptides discovered each year from 1970 to 2012. Data were obtained from the antimicrobial peptide database (*http://aps.unmc.edu/AP*) [8,9].

Many natural AMPs were isolated by chromatographic methods [5,6,7,32,33,34]. Based on the APD [8,9], the total numbers of natural AMPs discovered annually from 1970 to 2012 are plotted in Figure 2. While only a few AMPs were isolated during 1970–1984, up to 29 AMPs were reported between 1985 and 1992, and 41–82 between 1993 and 1999. In the 2000s, AMPs discovered reached ~100–150 per year. A sharp increase in such peptides in 2011 was primarily due to large-scale peptide discoveries from amphibians by using genomic and proteomic techniques [27,28,29]. Hundreds of the newly identified peptides listed in those papers have not been registered into the APD due to the lack of antimicrobial activity data. Our peptide registration practice [26] is supported by the observation that many of the predicted peptides synthesized for antimicrobial assays were found to be inactive or poorly active against the testing bacterial strains [28].

### 2.2. AMP Activity Annotated in the APD

It is now appreciated that natural AMPs could play multiple functional roles in host defense. As key effector molecules of innate immunity, they are produced to ward off invading microbes [31,35,36,37]. In insects, different types of AMPs can be expressed via different pathways in response to a variety of invading pathogens such as Gram-positive, Gram-negative bacteria, viruses, or fungi. Many of the insect defense elements appear to be conserved in mammals [38]. For example, the Toll pathway, initially found in insects, also exists in humans [39,40]. Some AMPs also have anti-cancer, anti-HIV, or spermicidal activities [7,31,32,33,34,41]. In addition, AMPs can modulate the immune system and elucidation of the mechanisms of immune modulation may provide yet another avenue for therapeutic peptide development [37,42,43]. The antimicrobial and chemotactic activities of AMPs are now annotated in the APD [9], allowing users to obtain a list of AMPs with defined activity. Table 1 lists the number of AMPs with various activities. In addition, our database also annotated anti-HIV peptides as well as antibacterial peptides that only active against Gram-negative or Gram-positive bacteria. These peptides can be used as the starting templates for designing peptides with desired properties.

**Table 1:** The number and abundant amino acids of antimicrobial peptides with defined biological activity ^1^.

Peptide activity	Peptide number	Abundant amino acids
Antibacterial	1768	L, G, S, K
Antiviral	158	C, G, S, R
Antifungal	777	C, G, S, K
Antiparasitic	48	C, G, S, K
Insecticidal	22	L, G, T, K
Spermicidal	9	A, G, T, K
Anticancer	145	C, G, S, K
Hemolytic	255	L, G, S, K
Chemotactic	41	L, G/P, S, K

^1^ Data obtained from the APD (*http://aps.unmc.edu/AP*) [8,9]. In the table, there are more peptides with antibacterial (1768) or antifungal (777) activity due to the common use of *E. coli*, *S. aureus*, and *C. albicans* in antimicrobial activity assays.

### 2.3. Calculations of AMP Parameters and Amino Acid Composition

**Figure 3:**
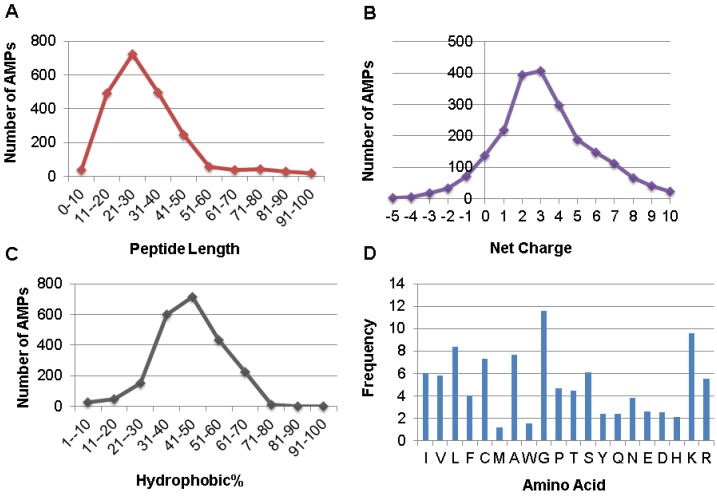
Distribution of AMPs as a function of peptide length (**A**), net charge (**B**), and hydrophobic% (**C**). The frequency of the 20 amino acids of AMPs in the APD (**D**). Data obtained from the APD (*http://aps.unmc.edu/AP*) [8,9].

The APD also enables users to obtain the parameters (e.g., length, net charge, hydrophobic content, and structure type) for a single peptide or a group of candidates [8]. The distribution of all the AMPs as a function of peptide length is given in Figure 3A. It is clear that 88% the AMPs possess 11–50 amino acids with the peak in the range of 21–30 residues. Assuming pH 7, the calculation of the net charge of an AMP involves acidic amino acids D, E, and basic R and K. A distribution of all the AMPs as a function of peptide net charge is given in Figure 3B. There are 86% of AMPs with a net charge between 0 and +7 (maxima at +2 and +3). The hydrophobic percentage is the ratio between the sum of the amino acids in the hydrophobic group and the total number of amino acids. A distribution of all the AMPs as a function of peptide hydrophobic% is given in Figure 3C. The majority of AMPs (89%) contain a hydrophobic% in the range of 31–70% (the maximum at 41–50%). In the APD database, the 20 standard amino acids are classified into four groups: hydrophobic (L, I, V, M, F, W, A, and C), PG (P and G), polar (S, T, N, Q, and Y), and charged (D, E, K, R, and H). To a large extent, the amino acid composition of AMPs determines peptide activity [44]. The APD is the first AMP database that allows users to calculate the amino acid composition profile for each peptide or a selected group of AMPs with a specific activity or property [8,9,26]. Figure 3D is such a profile for all the AMPs, where on average amino acids L, G, and K have the highest frequencies. Here we define the abundant amino acid as the one that has the highest frequency or percentage in each of the four amino acid groups defined above. The abundant amino acids for a group of AMPs with defined antimicrobial activity are listed in Table 1. For example, the abundant amino acids for the 1768 antibacterial peptides are L, G, S, and K (*i.e.*, identical to those in Figure 3D). However, C is the abundant hydrophobic residue for AMPs with antiviral, antifungal, or antiparasitic activity, indicative of the existence of a large population of cysteine-containing peptides in these activity groups. Because C/L, G, S/T, and K are consistently dominant in various activity groups in Table 1, they are preferred by nature in designing AMPs. In contrast, amino acids such as M, W, Y, Q, E, D, and H have the lowest frequencies on average. The biased use of amino acids in AMP design could be important for host defense since no active peptides were obtained in the 200 sequences assembled by choosing amino acids randomly [45].

The amino acids composition can be utilized to predict whether a natural peptide is potentially an AMP. This is the basis for programming the AMP prediction interface in the first version of the APD. The input peptide sequence is predicted as “rich” in certain amino acids, disulfide-linked (usually β-sheet structure), or α-helix [8]. Recently, Wang *et al*. showed that, among the multiple parameters used in peptide prediction, the contribution (weighting) of the amino acid composition accounts for 60% [46].

The amino acid composition profiles may also contain mechanistic information. For example, the amino acid profiles of bacterial lantibiotics and plant cyclotides are similar [26], although the sulfurs in these two families of peptides bridge side chains in a different manner: disulfide bonds in cyclotides but thioethers in lantibiotics. Interestingly, peptides from these two families can also share the same mechanism of action—both recognizing phosphatidylethanolamines (PEs) in membranes [47,48,49]. All these examples indicate that the amino acid composition calculated in the APD is an important feature of natural AMPs.

## 3. Peptide Discovery Based on the APD Database

Based on the APD database, various approaches have been developed for peptide discovery [50,51,52,53,54,55,56]. These methods, including linguistic model, database screening, *de novo* design, and template-based design, enabled the identification of potent peptides against human immunodeficiency virus type 1 (HIV-1) or methicillin-resistant *Staphylococcus aureus* (MRSA).

### 3.1. The Linguistic Model

Loose *et al.* developed a linguistic model for rational design of antibacterial peptides [51] based on the initial 525 peptide entries in the APD [8]. In this approach, the AMP sequences were regarded as a language. Over 700 sequence patterns (grammars) were identified from those AMPs and each grammar consisted of 10 amino acids. Just like the creation of a new sentence by grammar, they generated new AMPs by combining two grammars. This is reminiscent of the hybrid approach used by others to generate new peptides [57,58,59]. Two antibacterial peptides were identified for further optimization by first making the sequences more different from natural sequences and later more active by increasing cationic and hydrophobic amino acids. This method can clearly generate new sequences not yet found in nature.

### 3.2. Database Screening

For a particular natural peptide in the APD, antimicrobial activity assays are usually conducted with a limited number of microbial strains (typically *E. coli*, *S. aureus*, and *C. albicans*) and the strain which the peptide was expressed for or most active against might not have been uncovered. Hence, we hypothesized that the APD database (*i.e.*, a library of mainly natural peptides) contains useful candidates and can be screened to identify most potent peptide templates with desired activity. Ideally, the entire database with various templates should be experimentally screened. In reality, this is impractical due to many challenges in obtaining all these compounds at a reasonable cost and within a required time frame. Therefore, we utilized the database to help select a representative group of templates. Two separate screens were performed [52,53]. In one screening, our goal was to identify compounds against HIV-1. In another screening, we aimed to identify most potent peptides against MRSA USA300, a USA community-associated superbug. Database screening should be applicable to the identification of peptides with other types of activity as well.

#### 3.2.1. Identification of HIV-1 Inhibitory Peptides

The acquired immunodeficiency syndrome (AIDS) is one of the leading causes of death worldwide. The United Nations estimates that 1.5 to 1.9 million people died from HIV/AIDS worldwide in 2011 alone. Although the deaths dropped by 24% compared to those in 2005, continued efforts are needed to further reduce HIV-1 infections. In particular, effective HIV-1 vaccines are not yet available, making the development of topical microbicides desirable to prevent the HIV-1 transmission during sexual intercourses [41,60,61]. In collaboration with ImQuest Biosciences, we evaluated anti-HIV activity of 30 candidates using the established protocols (see ref. [56] for details). These peptides were selected from the APD database based on the following criteria: (1) length < 25 amino acid residues because shorter peptides are easier to synthesize in chemical labs; (2) net charge > 0, since anionic peptides tend to be inactive; (3) no cysteines, since the synthesis of peptides with multiple disulfide bonds are more time-consuming; (4) nontoxic to mammalian cells, because cytotoxicity is an undesired property; (5) not annotated as “synthetic” (*i.e.*, man-made peptides) in the database, as such peptides might have been patented by other laboratories; and (6) anti-HIV-1 activity unknown, since we want to identify new candidates. Of the 30 candidates, 11 peptides showed EC_50_ concentrations in the range of 0.63–7.1 μM [52]. The success rate of our database screening (37%) appeared to be high. The top six anti-HIV peptides are given in Table 2. These peptides originated from diverse sources: amphibians (ascaphin-8 and brevinin-2 related), insects (ponericin L2), fish (piscidin 1), and human engineered (DASamP1 and DASamP5). The names of the last two engineered peptides were derived from “database screened antimicrobial peptides 1 and 5). While DASamP1 (amino acid sequence: FFGKVLKLIRKIF-amide) is a variant of temporin-PTa [62], and DASamP5 (amino acid sequence: SLSRFLRFLKIVYRRAF-amide) is a variant of temporin-LTc [63]. Our screening identified new peptides with anti-HIV activity. As of March 2013, there are 92 anti-HIV peptides in our database. This only accounts for 4% of the database entries [9]. Thus, one may anticipate that more anti-HIV peptides could be identified in this manner.

**Table 2:** Top six potent antimicrobial peptides against *Staphylococcus aureus* USA300 LAC or HIV-1 identified from database screening ^1^.

Peptide Name	*S. aureus* USA300 (MIC, μM)	Peptide Name	HIV-1 (EC_50_, μM)
Ascaphin-8	3.1	Ascaphin-8	1.2
DASamP1	3.1	DASamP1	0.63
DASamP2	6.2	DASamP5	0.83
Lycotoxin I	3.1	Ponericin L2	1.4
Maculatin 1.3	6.2	Brevinin-2 related	1.65
Piscidin 1	3.1	Piscidin 1	2.1

^1^ Data obtained from refs [52,53]. See the text for additional information of these peptides.

#### 3.2.2. Identification of Anti-Staphylococcal Peptides

The spread of MRSA from the clinical setting to the communities is worrisome. In fact, the annual frequency of deaths from MRSA is rapidly increasing and has surpassed those caused by HIV/AIDS. Therefore, there is a need to develop new treatments to control MRSA. To identify new compounds against MRSA USA300 LAC, we also assayed the antibacterial activity of the same 30 peptides used for screening anti-HIV-1 peptides above [52]. Six peptides, including frog ascaphin-8, DASamP1, DASamP2, spider lycotoxin I, frog maculatain 1.3, and fish piscidin 1 (Table 2), were found to kill *S. aureus* with the minimal inhibitory concentrations (MIC) of 3.1–6.2 µM. DASamP2 (amino acid sequence: IKWKKLLRAAKRIL-amide) is a derivative of polybia-MPI [64]. To better gauge antimicrobial ability of these peptides, we also tested their antibacterial activity against Gram-positive *Bacillus subtilis*, and Gram-negative *Escherichia coli* and *Pseudomonas aeruginosa*. Aascaphin-8, DASamP2, lycotoxin I, and piscidin 1 are thus established as broad-spectrum AMPs that are active against *S. aureus* USA300, *B. subtilis*, *E. coli*, and *P. aeruginosa*. On contrary, maculatin 1.3 is only active against the two Gram-positive bacteria *S. aureus* USA300 and *B. subtilis*, but not the two Gram-negative bacteria *E. coli* and *P. aeruginosa*. DASamP1 appears to be species-specific since it is active only against *S. aureus*, but not other bacteria tested. Therefore, these six peptides with varying activity spectrum are useful templates for developing different antibacterial agents. For example, DASamP1 was able to prevent Staphylococcal biofilm formation in the catheter embedded in mice [53]. Considering the pain in replacing medical devices and the difficulty in treating biofilm infection, this short peptide may be developed into antimicrobial agents for topical use.

#### 3.2.3. Comparison of the Top Antibacterial and Antiviral Compounds

Table 2 lists the six most potent peptides against either MRSA or HIV-1. While ascaphin-8, DASamP1, and piscidin-1 are effective in inhibiting both MRSA and HIV-1, another three potent peptides differ, indicating that antiviral and antibacterial peptides identified in this way differ. We believe that the differences in antimicrobial targets could be the fundamental reason for obtaining a different set of potent templates from the same peptide library. For example, proline-rich AMPs are known to interact with bacterial heat shock proteins [65]. It is not surprising that drosocin, apidaecin IA, and metalnikowin in our database screening did not show any activity against HIV-1 since this virus does not have that protein target [52].

### 3.3. Database-Guided Design of Antimicrobial Agents

#### 3.3.1. *De Novo* Design of Anti-HIV Peptides

We also explored database approaches to designing antimicrobial compounds against HIV-1 [9,54]. Our initial attempt was a stepwise design based on the bioinformatics results from our database. In particular, we observed that AMPs from different kingdoms or families have distinct amino acid composition profiles [9,26,44]. When all the AMPs were statistically analyzed, amino acids L, G, and K were found to be abundant (Figure 3D). These three amino acids appear to contain sufficient information for peptide design, since we succeeded in designing an antibacterial peptide GLK-19, a 19-residue peptide containing only G, L, and K (Table 3). This peptide possessed good activity against *E. coli* (MIC 10 μM) but not HIV-1. Next, we intended to confer anti-HIV activity to this peptide template based on our database observation that arginines, rather than lysines, are preferred in antiviral peptides (Table 1) [8]. Indeed, GLR-19, an arginine analog of GLK-19, became HIV-1 inhibitory, although it lost activity against *E. coli* (Table 3).

The APD also suggests that antiviral peptides are rich in cysteines [8]. This finding inspired us to introduce a pair of cysteines into GLR-19 at varying positions along the polypeptide chain, leading to a series of 19-residue peptides with various loop sizes [54]. These GLRC peptides (Table 3) are made of only amino acids G, L, R, and C. Of the four such peptides, two showed higher anti-HIV activity than GLR-19. GLRC-2, the most potent peptide (EC_50_ 0.8 μM), has a disulfide bond between C4 and C16 (Table 3). It also has an inhibitory effect on herpes simplex virus type-2 (HSV-2) [54]. Our work illustrates that one can improve peptide activity step by step based on database-derived knowledge [8].

While GLRC-2 is most effective against HIV-1, the most potent antibacterial peptide is GLRC-3 (MIC 7.5 μM). It is evident that the best antibacterial and antiviral peptides possess different loop structures. Interestingly, the most potent anti-HIV peptide GLRC-2 is also most stable to the action of chymotrypsin (Table 3). One possible interpretation is that the polypeptide chain of GLRC-2 could be more compact than GLRC-3. 

**Table 3:** Database-guided design of anti-HIV peptides ^1^.

Peptide name	Amino acid sequence	Design strategy	EC_50_ (μM)	MIC (μM)	Stability (% left)	Ref.
GLK-19	GLKKLLGKLLKKLGKLLLK	G, L, K	>47.5	10	NE	[9]
GLR-19	GL**RR**LLG**R**LL**RR**LG**R**LLL**R**	K to R	4.4	>120	14.5	[52]
GLRC-1	G**C**RRLLGRLLRRLGRLL**C**R	C2-C18	>43.8	30	20.8	[54]
GLRC-2	GLR**C**RLGRLLRRLGR**C**LLR	C4-C16	0.79	30	63.3	[54]
GLRC-3	GLRRL**C**GRLGRRL**C**RLLLR	C6-C14	2.8	7.5	1.4	[54]
GLRC-4	G**C**RRL**C**GRLGRRL**C**RLL**C**R	C2-C18; C6-C14	30.7	60	9.7	[54]

^1^ The changed amino acids are in bold. Data taken from refs. [9,52,54]. EC_50_ and MIC are used as a measure of peptide potency against HIV-1 and *E. coli* K12. NE stands for “not evaluated”. For additional description of the table, see the text.

#### 3.3.2. *Ab Initio* Design of Anti-MRSA Peptides

It is also possible to design novel peptides based entirely on the APD [55]. We referred to this unique method as *ab initio* design to distinguish it from the *de novo* methods above. Our design comprises two major modules. The first module consists of an activity filter that allows us to obtain a list of peptides with desired activity (e.g., antibacterial, antifungal, antiviral, antiparasitic, or anticancer). To obtain compounds to kill *S. aureus*, we retrieved all AMPs with activity against Gram-positive bacteria as model peptides. The second module is composed of a series of parameter filters. In each step, we followed the most probable principle to derive the needed peptide parameters from the set of the model candidates. For example, a plot of peptide entries as a function of net charge showed that the largest peptide group in the 268 AMPs had a net charge of +1. This was translated into one lysine for the peptide. Likewise, the hydrophobic content of the peptide (61–65%) was also determined, leading to eight leucines for a 13-residue peptide. This peptide length was also determined by the database. These operations led to 1K, 2G, 2S, and 8L. Although such an amino acid composition could generate many different sequences, the possible candidates reduced dramatically once most probable peptide motifs came into consideration. In this way, we arrived at a unique peptide sequence dubbed DFTamP1, the first AMP designed based on the database filtering technology [55]. DFTamP1 possessed the activity and structure as designed. It is active against *S. aureus*, but not *E. coli*, *B. subtilis*, or *P. aeruginosa*. DFTamP1 is able to kill MRSA USA300 rapidly, suggesting membrane targeting. The membrane targeting is consistent with our observation [55] that the two versions of a similar peptide synthesized in all L- or d-amino acids are equally active [66,67]. These results set the stage for determining the active conformation in membrane-mimetic micelles by NMR spectroscopy (see below). DFTamP1 has indeed an amphipathic helical structure, ideal for membrane binding. In particular, the hydrophobic surface, consisting of a cluster of eight leucines, is critical for bacterial killing as substitutions of leucines with either valines or isoleucines led to poor activity and reduced solubility in the isoleucine case [55]. It seems that there is a good reason for nature to choose those abundant amino acids for AMP design (Table 1).

### 3.4. Template-Based Design and Optimization

It is feasible to design new AMPs based on known peptide templates collected in the APD. In one example, we succeeded in converting a non-toxic membrane anchor to an antibacterial peptide [50]. The starting peptide template commences with GLFD and can form a three-turn amphipathic helix covering residues 1–10 [68,69]. A search of the APD database led to a family of AMPs that share the N-terminal GLFD sequence with non-toxic membrane anchor. A representative member is the antimicrobial and anticancer peptide aurein 1.2 isolated from an Australian frog [70]. Aurein 1.2 with 13 residues was found to be entirely helical in membrane-mimetic organic solvents [70] or micelles [50]. In analog to aurein 1.2, the non-toxic bacterial membrane anchor gained antibacterial activity when D13 was changed to F13. It seems that tuning peptide length offers a mechanism for controlling peptide function.

As a second example, we also aimed to identify the minimal active region in a longer peptide. Human cathelicidin LL-37 is a 37-residue peptide starting with a pair of leucines. Our studies produced a library of model peptides with distinct antimicrobial activity. KR-12 is identified as the minimal antibacterial peptide corresponding to residues 18–29 of human LL-37. It is active against *E. coli* but not HIV-1 [56,71]. However, FK-13, a core antimicrobial peptide with one more phenylalanine at the N-terminus than KR-12 [72], is active against both HIV-1 and *E. coli*. Interestingly, the reverse sequence of FK-13 (retro-FK13) [73] is active against *E. coli*, but not HIV-1 [56]. These results underscore the importance of both F17 and sequence order for HIV inhibitory activity. While LL-23, the N-terminal fragment of LL-37, is inactive against HIV-1, GF-17, a central fragment of human LL-37 corresponding to residues 17–32 [72], is both bactericidal and virucidal [56,74]. Actually, GF-17 is the major antimicrobial peptide of LL-37. However, introduction of 1 to 2 d-amino acids to GF-17 abolished its anti-HIV activity [56]. In contrast, the same sequence remained antibacterial even after incorporation of three d-amino acids (*i.e.*, GF-17d3). NMR analysis revealed a novel non-canonical amphipathic structure for GF-17d3 [72]. Thus, anti-HIV activity of these LL-37 fragments requires a specific sequence as well as the helical structure [56]. Further extending the sequence of GF-17 to GI-20 (corresponding to residues 13–32 with the positions between I13 and G14 swapped) generated an anti-HIV peptide with the best therapeutic index [56,75]. Our observation is consistent with subsequent mechanistic studies using similar LL-37 fragments, which suggest the association of these fragments with HIV-1 reverse transcriptase *in vitro* [76]. In contrast, antibacterial activity of those LL-37 fragments can be attributed to membrane disruption, which does not require a defined peptide sequence or structure. This example, as well as the results from our database screening and *de novo* design discussed above, indicates the requirements of different peptides to target HIV-1 or pathogenic bacteria.

## 4. Differences in Binding Targets and Mechanisms of Action of AMPs

AMPs can be broadly classified into two families: cell surface targeting peptides and intracellular targeting peptides [26]. *Cell surface-targeting peptides*, including both membrane-targeting and non-membrane targeting peptides, can be further classified based on specific targets such as cell wall/carbohydrates, lipids/membranes, and proteins/receptors. Likewise, *intracellular targeting AMPs* can be further classified based on the specific target molecules (e.g., heat shock proteins, DNA, and RNA).

In the APD, 332 and 163 entries were annotated as AMPs only against Gram-positive and Gram-negative bacteria, respectively. This species specificity of AMPs to a large extent can be ascribed to the cellular differences (cell wall, membranes, and other molecules) between these two types of bacteria [77,78]. While lipopolysaccharides (LPS) are one important component in the outer membranes of Gram-negative bacteria, lipid II is an important precursor in the cell wall synthesis of Gram-positive bacteria. Our APD search identified 16 lipid-II targeting AMPs [9] that are able to inhibit cell wall synthesis [79,80,81]. These cell-wall active peptides may and may not be active against Gram-negative bacteria. If they do, they usually work by a different mechanism. For example, HNP-1 targets *S. aureus* cell wall but membranes of *E. coli* [82]. In line with this observation, two mirror-imaged peptides (*i.e.*, synthesized using either all l- or all d-amino acids) [66,67] showed identical activity against *E. coli* but not *S. aureus*. Interestingly, HNP-1 binds to HIV-1 gp-120 glycoprotein (a lectin), but shows inhibitory effects after HIV-1 entry [83,84]. Thus, the demonstration of the association of an AMP with certain molecule from a pathogen *in vitro* may not necessarily mean that it is also responsible for *in vivo* killing [26].

The fundamental differences between bacteria and human cells, at least at the membrane level, are clearly significant from the standpoint of cell selectivity of AMPs. While bacterial membranes are rich in anionic lipids such as phosphatidylglycerols (PGs), human cell membranes are dominated by phosphocholines (PCs) and also contain cholesterol [85,86,87,88]. This explains why cationic AMPs are emphasized in the current research because they have preference for anionic bacterial membranes.

There are numerous biophysical techniques that can lead to useful mechanistic information. Confocal microscopy can be used to follow the location of a fluorescent tagged peptide in cells. Surface or intracellular locations can be judged directly from the images. Using this technique, magainin 2 was found to be located on the bacterial surface to form a pore, while buforin II was found to be within the cell. Further *in vitro* studies by gel retardation demonstrated its association with DNA [89]. For this cell-penetrating peptide, DNA binding could be important for killing.

The mode of peptide-membrane interactions appears to play an important role in bacterial killing [90]. Lipid vesicles are useful models to provide insight into peptide-membrane interactions [74,91,92]. While magainin 2 induced lipid flip-flop and caused dye leakage in lipid vesicles, buforin II did not [92]. In particular, the proline in buforin II is critical for membrane penetration [93]. Based on this observation, it may not be surprising that proline-rich peptides such as drosocin, pyrrhocoricin, and apidaecin can also cross the membranes of Gram-negative bacteria and associate with heat shock proteins as shown by mass spectrometry, Western blot, and fluorescence polarization experiments [65]. However, these proline-rich peptides appeared to bind to LPS first. Consistent with protein binding, a peptide analog of pyrrhocoricin synthesized in all d-amino acid was inactive [65]. Electron microscopy (EM) is also a powerful tool that can be used to view whether the cell is intact, damaged or lysed [55,94,95]. Indicator molecules can be used to report membrane permeation or disruption by flow cytometry [96]. For example, GF-17, the major antimicrobial region of human antimicrobial peptide LL-37, can disrupt bacterial membranes based on the fluorescence increase due to the association of non-permeable dye with DNA [74].

Human LL-37 is an important cationic defense peptide that has been extensively studied by various biophysical techniques. To provide insight into the action of the peptide, especially on membranes, polarized FT-IR and solid-state NMR techniques were utilized to indicate a surface location of the peptide on lipid bilayers [97,98]. Interestingly, Huang detected two states for LL-37 using oriented circular dichroism (OCD) and neutron in-plane scattering [99]. In the first state, LL-37 is parallel to the membrane surface as also observed by other techniques; in the second state, the peptide is perpendicular to the membrane corresponding to a pore formation state. A peptide orientation parallel to the membrane surface would support the carpet model [87,88], while an orientation perpendicular to the membrane surface suggests pore formation [99]. However, such results obtained by different techniques under different conditions are not always easily reconciled. In addition, prior to our work, outstanding structural questions remain. Which region of LL-37 is involved in membrane binding? Is there any basic residue important for this binding? What is the basis for cooperative binding of LL-37 to LPS? We addressed these questions by high-resolution NMR studies (see below) [100].

## 5. Structural Annotation, Classification and Determination of AMPs

### 5.1. Structural Annotation and Classification

**Figure 4:**
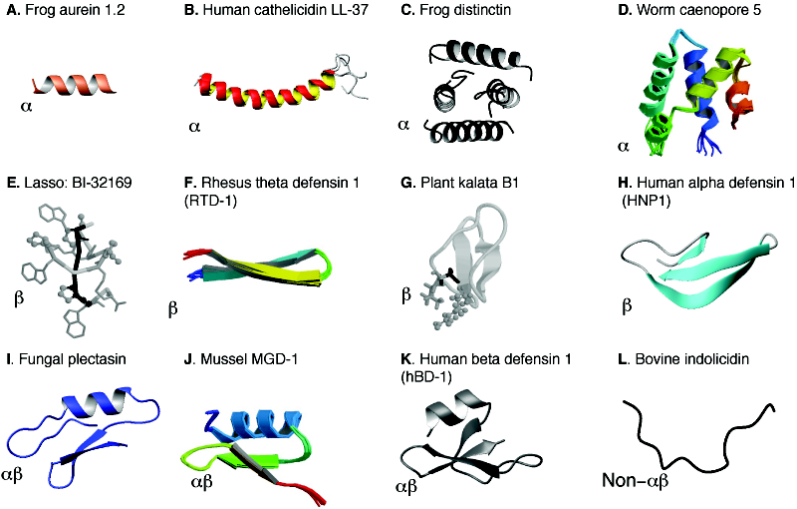
Structural diversity and classification of natural antimicrobial peptides. The known structures of AMPs are classified into four families (α, β, αβ, and non-αβ) [26] and the structural family for each peptide is indicated at the left bottom corner of each panel. The PDB IDs and references for these structures are: (**A**) 1VM5 [50] for aurein 1.2 in complex with SDS micelles; (**B**) 2K6O for human cathelcidin LL-37 in complex with SDS micelles [71]; (**C**) 1XKM for amphibian distinctin in water [114]; (**D**) 2JS9 for caenopore-5 [115] from *Caenorhabditis elegans*; (**E**) 3NJW for BI-32169 as a representative lasso structure [116]; (**F**) 2LYF for θ-defensin RTD-1 [117,118]; (**G**) 1KAL for plant kalata B1 [119]; (**H**) 3GNY for human α-defensin 1 (or human neutrophil peptide-1; HNP-1) [120]; (**I**) 1ZFU for fungal plectasin [80]; (**J**) 1FJN for mussel MGD-1 [121]; (**K**) 1E4S for human beta defensin 1 (HBD-1) [122]; and (**L**) 1G89 for bovine indolicidin [123]. Structural coordinates were obtained from the RCSB Protein Data Bank (PDB) [124].

Once the molecular target has been elucidated by various experiments above, one feels like to examine in details how the AMP in action looks like. While FT-IR, circular dichroism (CD), and fluorescence spectroscopies only provide low-resolution information, high-resolution techniques such as NMR spectroscopy and X-ray diffraction are able to reveal the atomic details of a peptide in water (*e.g*. distinctin and defensins), binding to membranes or in complex with other molecular targets. Structural information for each AMP is also annotated in the APD [8]. It is clear that AMPs can adopt a variety of structural scaffolds (Figure 4) [100,101,102]. By following the link for each peptide entry in the APD, one can view the 3D structure of the peptide if it is deposited in the Protein Data Bank. In March 2013, there were 288 peptides with known 3D structures in the APD database. Of these, 260 were solved by NMR and 28 by X-ray crystallography. It is evident that more AMP structures are determined by NMR spectroscopy than the X-ray method [8,9]. A possible reason for this may be the difficulty in obtaining crystals because many linear AMPs are disordered in water and only become ordered in the presence of membrane-mimetic models. For solution NMR studies, micelles are usually preferred, although organic solvents are also used. Rapid tumbling of small micelles in solution averages out chemical shift and dipolar coupling anisotropies, leading to high-resolution NMR spectra for structural determination. The commonly used micelle models are sodium dodecylsulfate (SDS) and dodecylphosphocholine (DPC) [75,103,104,105,106,107]. While dihexanoyl phosphatidylcholine (DHPC) is occasionally utilized [108,109], dioctanoyl phosphatidylglycerol (D8PG) should better mimic bacterial membranes than SDS [110,111,112,113]. This D8PG model is unique because arginine side chain amide signals are well resolved, allowing for the detection of arginine-PG interactions by 2D NMR spectroscopy [75]. A more complete description of membrane models and NMR methods can be found elsewhere [113].

In the APD, various three-dimensional structures [50,71,114,115,116,117,118,119,120,121,122,123] are classified into four families: α, β, αβ, and non-αβ (see Figure 4) based on the presence or absence of secondary structures such as α-helix and β-sheet [26]. The α family consists of AMPs with α-helical structures (Figure 4, Panels A–D). Only a few peptides (e.g., distinctin) possess a helical structure even in water due to the stabilization of disulfide bonds (Panels C and D). Many AMPs are only helical in complex with bacterial targets such as membranes (Panels A and B). The β family is characterized by the formation of β-sheet structures (panels E-H). The αβ family contains both α-helical and β-sheet structures (panels I-K). Multiple disulfide bonds in these molecules are responsible for the observed structure in aqueous solution. Examples are various defensin-like molecules from fungi, plants, mussels, monkeys, and humans (Figure 4). Although there are few examples, the non-αβ family consists of neither α-helices nor β-sheet structure (panel L). A more complete and updated list of AMPs from each structural family can be obtained in the APD database [8,9].

### 5.2. Structural Determination of Database-Designed Peptides by the Improved 2D NMR Method

Some types of AMPs are rich in amino acids such as prolines and histidines. These peptides could pose problem for structural determination by the traditional proton-only 2D NMR method. One alternative approach is to use the improved 2D NMR, which was first demonstrated in 2005 [50]. The major experiments for the traditional and improved 2D NMR methods are listed in Table 4. It is clear that additional experiments are conducted in the case of the improved 2D NMR method. In particular, heteronuclear correlated experiments are recorded at natural abundance to extend the measurements beyond ^1^H. Our measurements of ^13^C and ^15^N chemical shifts at natural abundance are cost effective because one can avoid the cost in producing isotope labeled polypeptides [100]. In the following, we use two recent examples to illustrate the merits of the improved 2D NMR method for structural determination of small AMPs.

**Table 4:** Key experiments for traditional and improved 2D NMR methods.

Nucleus	Traditional 2D NMR method [103]	Improved 2D NMR method [50]
^1^H	TOCSY, DQF-COSY, and NOESY	TOCSY, DQF-COSY, and NOESY
^13^C		Natural abundance (^1^H, ^13^C) HSQC
^15^N		Natural abundance (^1^H, ^15^N) HSQC

**Figure 5:**
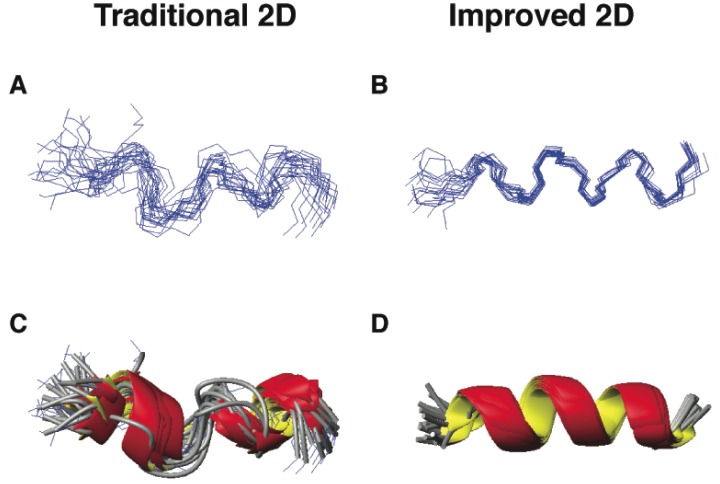
Three-dimensional structures of DFTamP1 determined by traditional (panels A and C) [103] and improved 2D NMR methods (panels B and D) [50]. Shown are ensembles of backbone structures (A and B) and ribbon diagrams (C and D). All the NMR data were recorded on a 600-MHz NMR spectrometer using a 2 mM peptide in complex with 40-fold deuterated sodium dodecyl sulfate at 25 °C and pH 5.4. Structures (panels A and C) were determined as described previously [55] and the structures without the use of chemical shift-derived angle restraints (panels B and D) were calculated in this study.

The first example is DFTamP1, an anti-MRSA peptide designed in 2011 based on our database [55]. Of the 13 amino acids in this peptide, there are eight leucines (61.5%), which appeared in similar spectral regions. The spectral overlap made some proton cross peaks unresolved, leading to a low-resolution structure (Figure 5, panels A and C; Wang, unpublished data). To improve spectral resolution, we recorded heteronuclear single quantum coherence (HSQC) spectra at natural abundance (only 1.1% for ^13^C and 0.36% for ^15^N). Both ^13^C and ^15^N heteronuclei have much broader chemical shift ranges than ^1^H, making it possible to measure different carbon or nitrogen chemical shifts even when the proton chemical shifts are similar. The inclusion of heteronuclear chemical shifts derived angle restraints into structural calculations further defined the structural ensemble, leading to consensus helical structures (Figure 5, panels B and D).

**Figure 6:**
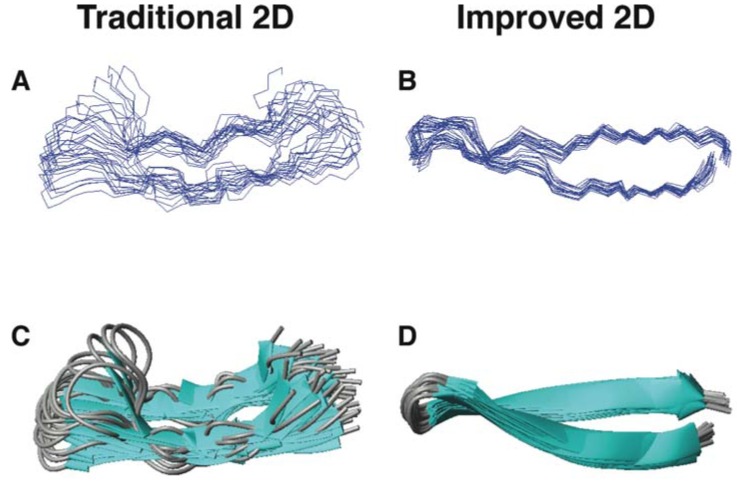
Three-dimensional structures of RTD-1 determined by traditional (panels A and C) and improved 2D NMR methods (panels B and D) [117,118]. Shown are ensembles of backbone structures (A and B) and ribbon diagrams (C and D). Data were obtained from the Protein Data Bank (PDB entries: 1HVZ and 2LYF) [124].

As a second example, another group also utilized the improved 2D NMR [50] to re-determine the structure of θ-defensins such as RTD-1 in 2012 [117]. RTD-1 is the first circular AMP found in mammals [32]. This peptide is rich in both arginines and cysteines (sequence: GFCRCLCRRGVCRCICTR). As anticipated, the new structure (Figure 6B,D) is better defined by using the improved 2D NMR method than that (Figure 6A,C) determined previously by the traditional proton 2D NMR spectroscopy [118]. Craik and colleagues discussed that “Earlier structure calculation on RTD-1 and HTD-2 did not have access to the ^13^C and ^15^N HSQC information, leading to the suggestion that the θ-defensins were flexible”. In the abstract, they concluded that “the θ-defensins are more rigid and structurally defined than previously thought” [117]. The rigid structure of circular θ-defensins is stabilized by three pairs of parallel disulfide bonds, leading to a ladder-like structure.

These two examples in Figure 5, Figure 6 represent AMPs from different structural families: α-helix for DFTamP1 [55] and β-sheet for RTD-1 [117,118]. Furthermore, these structures were determined in different environments. While RTD-1 was determined in an aqueous solution, DFTamP1 was determined in the presence of membrane-mimetic SDS micelles. Although these two structures were determined by different laboratories, the outcomes are remarkably similar—both pointing to the advantage of the improved 2D method [50] that gave more precisely defined peptide structures. In both cases, the TALOS program [125] was utilized to convert a set of heteronuclear chemical shifts (^1^H, ^15^N, ^13^Cα, and ^13^Cβ) into backbone angles for structural refinement. Protein dynamics analysis confirmed that the well-defined structural regions are indeed rigid (*i.e.*, less mobile) [71,117,126]. These examples indicate that the improved 2D NMR is able to generate the correct structures for these small AMPs with a biased amino acid composition, thereby avoiding the publication of misleading structure and dynamics.

### 5.3. Beyond 2D NMR

Although the improved 2D NMR method [50] led to impressive results (Figure 5, Figure 6), it is important to be aware of its limitations. When there is too much spectral overlap, it is necessary to use 3D heteronuclear NMR techniques. This is exactly the case with the structural determination of human cathelicidin LL-37 in membrane-mimetic micelles. Isotope labeling of LL-37 was achieved by expressing the peptide in *E. coli* in a chemically defined minimal medium containing ^15^N-NH_4_Cl and ^13^C-glucose followed by formic acid cleavage and chromatographic purification [127,128]. This isotope-labeled LL-37 enabled the collection of 3D triple-resonance NMR experiments [129], where the crowded proton cross peaks were resolved by separating them onto different 2D planes along the third ^15^N or ^13^C dimension. The excellent spectral resolution enabled us to determine the LL-37 structure to high quality [71]. Our high-quality structure of human LL-37 in membrane-mimetic micelles is entirely consistent with backbone dynamics (ps-ns time scale) of the polypeptide chain, further validating the structure. In addition, we found essentially the same structure for LL-37 in SDS and D8PG micelles. We also obtained a similar structure for human innate peptide LL-23 in SDS, DPC and D8PG micelles [107]. These results suggest that it is spectral resolution, rather than the micelle model, that led to the differences in the two structures of LL-37 determined by 2D [130] and 3D NMR [71] techniques.

Our high-quality LL-37 structure provides a solid basis for understanding the interactions of this important human cationic peptide with anionic bacterial membranes. The long amphipathic helix covering residues 2–31 of LL-37 (Figure 4B) is required for interactions with both LPS (one of the major component in Gram-negative bacterial outer membranes) and PGs (major anionic lipids in bacterial inner membranes) [71]. Our introduction of the D8PG model [110,111,112,113] allowed for the first detection of aromatic-PG and arginine-PG interactions by solution NMR spectroscopy, indicative of the importance of both electrostatic and hydrophobic interactions between the cationic peptide and anionic lipids [112]. Interestingly, the same arginine R23 (not all arginines) that contacts PGs is also the most important one for antibacterial activity, membrane permeation and lipid clustering [74]. A recent study suggests that R23 is also important for interactions with DNA quadruplex [131]. The cooperative binding of human LL-37 with LPS observed earlier by Lehrer and colleagues [132] can also be understood based on the high-quality 3D structure of LL-37—a unique serine at position 9 splits the hydrophobic surface of the long amphipathic helix into two domains [71,107]. These two domains can work together to better bind to LPS. Our high-quality structure also serves as a model to understand the structure-activity relationship of homologous primate cathelicidins. For example, a helical bend was mapped to residues 14–16 in the LL-37 structure. In the same region of homologous cathelicidins, as many as three glycines are possible [71]. Our studies also provide insight into the LL-37 oligomerization [97]. First, the elution times of a series of proteins on a size-exclusion column suggest a tetramer. Second, NMR data suggest the involvement of residues 2–36 in tetramer formation [100,127].

AMPs can use a variety of molecular targets and not all these targets are amenable to NMR studies. Under these circumstances, one may consider other biophysical techniques such as X-ray diffraction. For example, the structure of a Pro-rich peptide in complex with heat shock protein has recently been determined [133]. This structure laid a solid foundation for structure-based design of novel antibiotics.

In summary, this section emphasizes the importance of choosing a proper method for structural determination of AMPs to avoid structural artifacts. In many cases where the spectra are well resolved, the traditional 2D NMR method [103] is sufficient. For example, we found it redundant to include heteronuclear chemical shift-derived restraints into the structural calculations of aurein 1.2 (Figure 4A) because of many well-resolved proton NMR restraints [50]. However, under certain circumstances such as those amino acid-rich peptides in Figure 5, Figure 6, the improved 2D NMR method [50] is no doubt important to produce a correct structure. We recommend the use of the improved NMR method for structural determination of small AMPs because it helps improve and validate the structure [113]. Note that the high-quality structure of LL-37 was only made possible via 3D NMR studies, because 2D NMR was insufficient in this case [71,72]. Advanced NMR and/or X-ray techniques should be considered for more complicated structural problems involving AMPs.

## 6. Concluding Remarks

Nature keeps inspiring us in discovering new medicine from the ancient time to the modern era [134,135]. Antimicrobial peptides have been recognized as critical defense molecules to ward off invading pathogens thanks to the pioneering work of the forerunners who took tremendous efforts in isolating and characterizing many natural AMPs in the classic way [5,6,7,31,35,36,42,43]. Several databases have been built to manage this information [8,9,10,11,12,13,14,15,16,17,18,19,20,21,22,23,24,25,26]. The antimicrobial peptide database [8,9] is a leading resource for nomenclature, classification, information search, statistical analysis, prediction and design of AMPs. The APD database has been expanded substantially as a consequence of regular update and continued developments. The growing amount of information and extensive annotation increases the possibility of identifying therapeutic molecules of interest. Based on the APD, we have demonstrated various approaches for peptide discovery [52,53,54,55,56,136]. Our database screening [52,53] appears to have a higher success rate than combinatorial library screens in identifying antimicrobials [137,138,139]. This may not be surprising because AMPs have been optimized in nature as host defense molecules, whereas randomness has to be introduced in artificial libraries even though the amino acids used are biased. An outstanding feature of the APD database is that it is composed of multiple information filters that can be assembled in numerous ways to extract the needed information. Such a database feature enabled us to conduct both step-by-step database-guided design of anti-HIV peptides and *ab initio* design of anti-staphylococcal peptides based on the database filtering technology. The sequence space of the designed peptides can be further explored via sequence shuffling or amino acid mutations [51,52]. Alternatively, the identified peptide may be further optimized by screening a synthetic peptide library [56] or conducting *in silico* screening [45]. *In silico* screening is preferred as it is cost effective. However, the scope of its applications may be limited because each program only shows peak performance on sequences that resemble those being trained. Also, it remains experimentally validated whether the inactive sequences in the training set are truly inactive. Therefore, one can anticipate that experimental screening and validation of peptide candidates will continue, although it can be time consuming, labor extensive, and expensive.

Recently, there is a growing interest in AMP prediction. This is because it is cheaper to identify AMPs from the genome than to isolate AMPs from natural sources. The APD programmed a peptide calculation and prediction interface in 2003 [8]. Our database data set is frequently used in developing other prediction methods [140,141,142,143,144]. Some known prediction protocols are also programmed into recent databases such as CAMP and DAMPD [10,11]. In AMPer, the information from both the mature peptide and precursor sequences were utilized [145]. While it is pre-mature to correctly predict every possible AMP gene from a genome, such prediction programs may be helpful to locate potential genes followed by experimental validation. Here the prediction can be based on the sequence of conserved modifying enzymes or the genomic context depending on the amount of information involved in the program [18]. This approach plays an important role in identifying novel bacteriocins in bacterial genome [146]. There are also programs that predict AMPs by scanning known protein sequences [147]. Whether the predicted peptides are actually generated for biological defense is not clear.

Both peptide screening and *de novo* design suggest different requirements for AMPs depending on the target pathogens (or more exactly molecular targets). While sequence order and the helical structure of LL-37 fragments are less important in killing *E. coli* due to membrane targeting [74], such features are essential in inhibiting HIV-1 due to interactions with the viral reverse transcriptase [76]. It is evident that peptide length is also an important factor in determining peptide activity spectrum [50,56]. While the full length LL-37 is implicated in cancer and became less effective against parasites or superbugs such as MRSA USA *in vitro*, its central fragments are effective in killing cancer cells or pathogens [74,148,149]. In addition, one can also improve cell selectivity by maximizing bacterial targeting and minimizing toxic effects on human cells [150,151,152]. In our database design, it is possible to insert a selectivity filter after activity and parameter filters [55]. The basis for this design has been laid since the APD annotated the cytotoxicity data of AMPs from the first version [8]. Juretic and colleagues designed selective glycine-rich peptides based on a library of helical AMPs from amphibians [85]. As we proposed previously, modulating peptide hydrophobicity provides a general approach for improving peptide cell selectivity, although there are various methods to achieve incoherent hydrophobic packing [72].

Whatever the sources of the peptide templates (isolation from natural sources, *de novo* peptide design, or *in silico* prediction from the genome), they are all subject to stability tests as a requisite for practical use. The differences in amino acid composition and structural scaffold in the case of database-designed anti-HIV and anti-MRSA peptides required different strategies to improve peptide stability to proteases. We achieved this by introducing a disulfide bond in the case of anti-HIV peptides (Table 3) [54], and non-standard d-amino acids [55] in the case of database designed peptides. Our design was mainly guided by the APD database [9,136], which also annotates nature’s post-translational modification strategies for AMPs (for a review, see ref. [153]). These chemical modification strategies should inspire peptide engineering to make natural AMPs more druggable. It is useful to point out that peptide stability and activity are not necessarily always programmed into the same molecular form. While the disulfide bond of distinctin is required for stability (peptide storage) but not activity [114], human beta defensin-1 (HBD-1) is more active after disruption of the disulfide bonds [154]. For those AMPs with a stable scaffold that is already protease-resistant (e.g., cyclotides), much effort is now spent on conferring the desired activity to the molecule by sequence mutation or grafting [155,156,157]. A careful structural determination is highly recommended because high-quality structures form the basis for us to interpret biology correctly and to conduct rational peptide design more effectively. In the end, it is useful to mention two nature’s antibiotics. Gramicidin, with a mixture of L- and D-type amino acids, is the first peptide antibiotics approved for clinical use [2]. Daptomycin, a cyclic anionic lipopeptide discovered in the 1980s, was approved by the FDA in 2003 to treat skin infection [158,159,160]. These successful peptides with multiple chemical modifications should also inspire the development of natural compounds into a battery of new therapeutic molecules.
